# Supplementary material for: ANXA11 rs1049550 Associates with Löfgren’s Syndrome and Chronic Sarcoidosis Patients
Source: Cells. 2022 May 5;11(9):1557. doi: 10.3390/cells11091557 (PMC9101321; doi:10.3390/cells11091557)
Supplement: Supplementary file 1 [file cells-11-01557-s001.zip › cells-1650145-supplementary.pdf]

Supplementary Table S1. Studies reporting an association of *ANXA11* rs1049550 with sarcoidosis disease phenotypes.

**Study**	**T allele Frequency**			**OR, 95% CI, *p* Value ***		
	**Control**	**Stage (0)-I**	**Stage II-IV**	**Stage (0)I vs. Controls**	**Stage II-IV vs. Controls**	**Stage II-IV vs. Stage (0)-I**
**Mrazek et al., 2011 [5]**	0.42 (*n* = 254)	0.42 (*n* = 110)	0.30 (*n* = 117)			0.61 (0.41–0.85) *p* = 0.01
**Levin et al., 2013 * [6]**	0.18 (*n* = 893)		Stage IV: (*n* = 188)		stage IV: 0.85, 0.62–1.16, *p* = 0.31[§,†]	
**Feng et al. 2014 [8]**	0.40 (*n* = 418)	0.28 (*n* = 196)	0.37 (*n* = 167)	0.56, 0.43–0.73, *p* = $1\cdot10^{-5}$	0.85, 0.65–1.10, *p* = 0.22	1.51, 1.11–2.07, *p* = 0.01
**Mirsaeidi et al. 2016 * [10]**		0.10 (*n* = 35)	Stage IV: 0.26 (*n* = 17)			3.24, 1.09–9.65, *p* = 0.03
**Sikorova et al., 2020 [9]**	*n* = 100	*n* = 29	*n* = 68	*0.69, p = 0.29*	0.53, 0.33–0.84, *p* = 0.01[†]	
	**Control**	**Resolving**	**Chronic**	**Resolving vs. Controls**	**Chronic vs. Controls**	**Chronic vs. Resolving**
**Li et al., 2010 [4]**	0.45 (*n* = 313)	0.35 (*n* = 117)	0.34 (*n* = 176)	0.65, 0.48–0.89, *p* = 0.007	0.62, 0.47–0.81, *p* = $5\cdot10^{-4}$	0.95, 0.67–1.34, *p* = 0.76
**Levin et al., 2013 [#] [6]**	0.18 (*n* = 893)	*n* = 304	*n* = 650	0.82, 0.64–1.06, *p* = 0.13[§,†]	0.79, 0.65–0.95, *p* = 0.02[§,†]	
**Morais et al., 2013 [7]**	0.45 (*n* = 197)	0.34 (*n* = 86)	0.40 (*n* = 62)	0.62, 0.43–0.91, *p* = 0.01	0.83, 0.55–1.25, *p* = 0.37	1.33, 0.82–2.14, *p* = 0.24
**Karakaya et al.**	0.41 (*n* = 363)	0.32 (*n* = 142)	0.26 (*n* = 113)	0.66, 0.50–0.89, 0.005	0.51, 0.36–0.70, *p* = $4\cdot10^{-5}$	0.76, 0.52–1.12, 0.65
	**Control**	**Löfgren's Syndrome**	**Non-Löfgren's Syndrome**	**Löfgren's Syndrome vs. Controls**	**Non-Löfgren's Syndrome vs. Controls**	**Non-Löfgren's Syndrome vs. Löfgren's Syndrome**
**Morais, 2013 [7]**	0.45 (*n* = 197)	0.36 (*n* = 55)	0.32 (*n* = 145)	0.70, 0.45–1.08, *p* = 0.11	0.57, 0.42–0.78, *p* = $5\cdot10^{-4}$	0.81, 0.51–1.29, *p* = 0.38
**Karakaya et al.**	0.41 (*n* = 363)	0.33 (*n* = 149)	0.26 (*n* = 113)	0.69, 0.52–0.92, *p* = 0.01	0.51, 0.36–0.70, *p* = $4\cdot10^{-5}$	0.73, 0.50–1.07, *p* = 0.11
**Mrazek, 2011 [5]**	TT frequency: 0.15 (*n* = 254)	TT frequency: 0.21 (*n* = 39)	TT frequency: 0.07 (*n* = 147)			0.31, 0.11–0.84, *p* = 0.02[†]

* Odds ratio (OR), 95% confidence interval (CI) and p-values are calculated from the data provided in the original articles.

[#] Studypopulation: African Americans. For correct presentation the C and T allele are switched.

[§] The original article states that the additive genetic model was used to estimate the OR, and the OR is adjusted for sex and percent African ancestry.

[†] Original data were not available, values are copied from the original article.

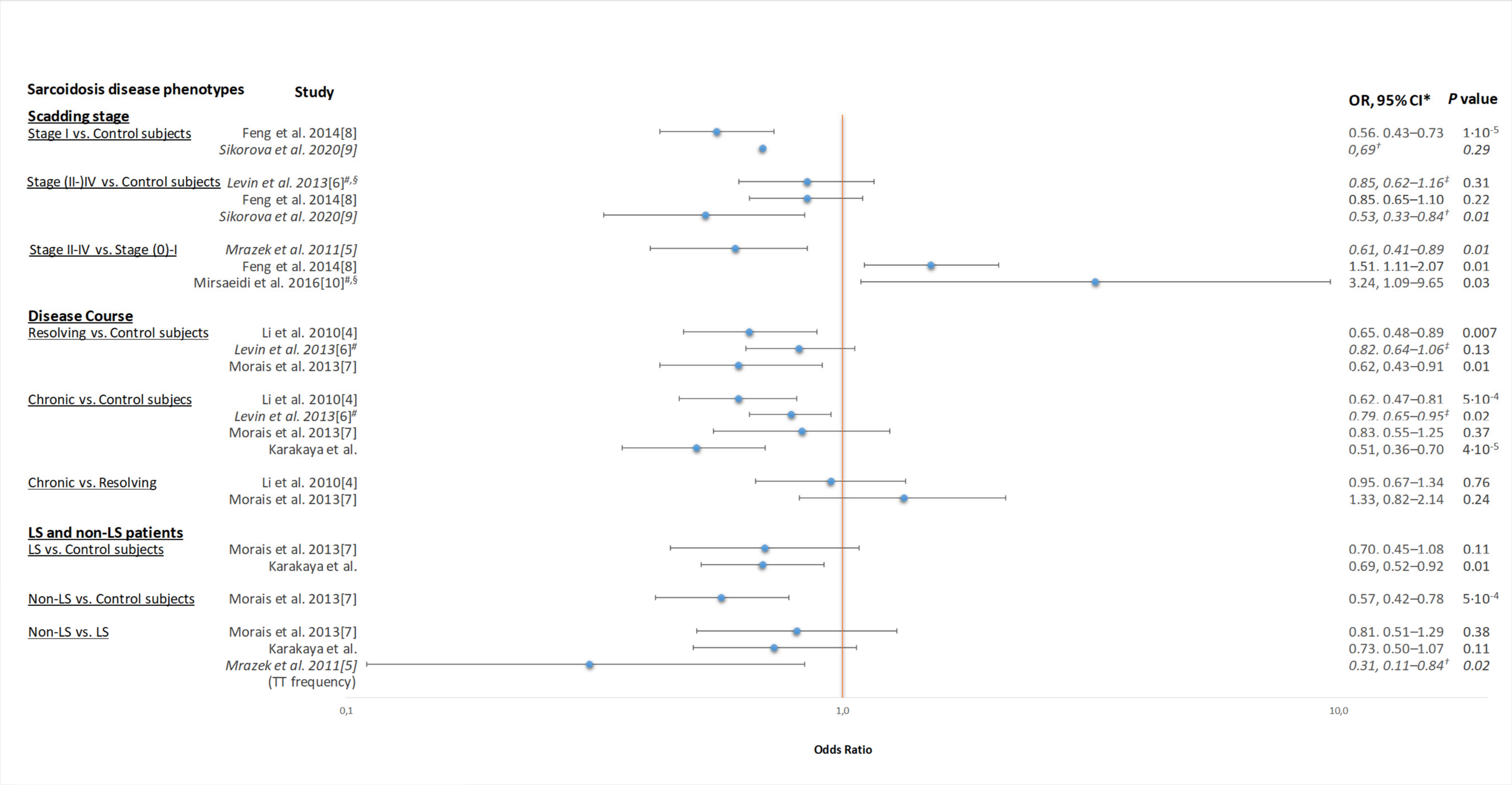

Supplementary Figure S1. Forest plot of the results for *ANXA11* rs1049550 T allele associations phenotypes of sarcoidosis.

* Odds ratio (OR), 95% confidence interval (CI) and p-values are calculated from the data provided in the original articles.

† Original data were not available, values are copied from the original article.

\# Studypopulation: African Americans. For correct presentation the C and T allele are switched.

§ only Scadding stage IV patients were analyzed.

‡ The original article states that the additive genetic model was used to estimate the OR, and the OR is adjusted for sex and percent African ancestry.
